# Integration of ER protein quality control mechanisms defines **β** cell function and ER architecture

**DOI:** 10.1172/JCI163584

**Published:** 2023-01-03

**Authors:** Neha Shrestha, Mauricio Torres, Jason Zhang, You Lu, Leena Haataja, Rachel B. Reinert, Jeffrey Knupp, Yu-Jie Chen, Gunes Parlakgul, Ana Paula Arruda, Billy Tsai, Peter Arvan, Ling Qi

**Affiliations:** 1 Department of Molecular and Integrative Physiology, University of Michigan Medical School, Ann Arbor, Michigan, USA.; 2 Division of Metabolism, Endocrinology & Diabetes, Department of Internal Medicine, University of Michigan Medical School, Ann Arbor, Michigan, USA.; 3 Department of Molecular, Cellular, and Developmental Biology, School of Literature, Science, and the Arts, University of Michigan, Ann Arbor, Michigan, USA.; 4 Department of Cell and Development Biology, University of Michigan Medical School, Ann Arbor, Michigan, USA.; 5 Department of Nutritional Sciences and Toxicology, University of California Berkeley, Berkeley, California, USA.; 6 Chan Zuckerberg Biohub, San Francisco, California, USA.

**Keywords:** Cell Biology, Metabolism, Autophagy, Diabetes, Protein misfolding

## Abstract

Three principal ER quality-control mechanisms, namely, the unfolded protein response, ER-associated degradation (ERAD), and ER-phagy are each important for the maintenance of ER homeostasis, yet how they are integrated to regulate ER homeostasis and organellar architecture in vivo is largely unclear. Here we report intricate crosstalk among the 3 pathways, centered around the SEL1L-HRD1 protein complex of ERAD, in the regulation of organellar organization in β cells. SEL1L-HRD1 ERAD deficiency in β cells triggers activation of autophagy, at least in part, via IRE1α (an endogenous ERAD substrate). In the absence of functional SEL1L-HRD1 ERAD, proinsulin is retained in the ER as high molecular weight conformers, which are subsequently cleared via ER-phagy. A combined loss of both SEL1L and autophagy in β cells leads to diabetes in mice shortly after weaning, with premature death by approximately 11 weeks of age, associated with marked ER retention of proinsulin and β cell loss. Using focused ion beam scanning electron microscopy powered by deep-learning automated image segmentation and 3D reconstruction, our data demonstrate a profound organellar restructuring with a massive expansion of ER volume and network in β cells lacking both SEL1L and autophagy. These data reveal at an unprecedented detail the intimate crosstalk among the 3 ER quality-control mechanisms in the dynamic regulation of organellar architecture and β cell function.

## Graphical abstract

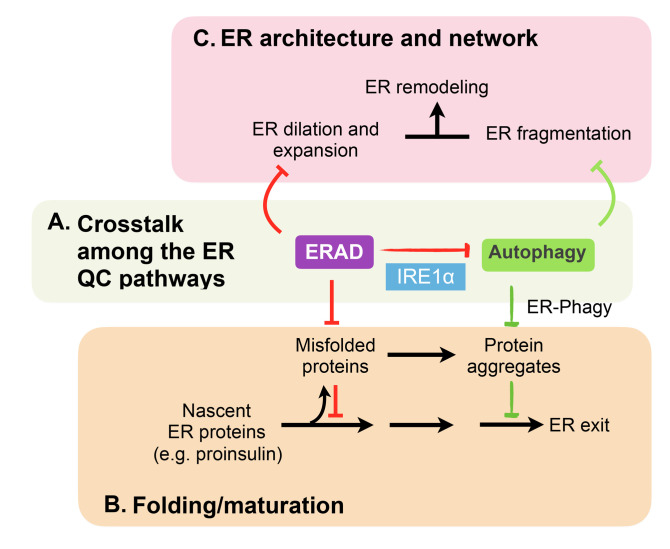

## Introduction

Pancreatic β cells are professional secretory cells capable of synthesizing thousands of proinsulin molecules per second in the ER (1). ER architecture and proteostasis are both highly dynamic and related; indeed, both features are tightly regulated via a balanced act of protein biosynthesis and degradation in response to environmental cues and nutrient status (2–4). A decline of proteostasis in the ER likely contributes to β cell dysfunction (including both dedifferentiation and cell death) in diabetes (5). As cytoplasmic organelles exist in a 3D environment, a major challenge is to visualize these 3D subcellular structures and delineate their dynamics. Three highly conserved quality-control mechanisms, the unfolded protein response (UPR), ER-associated degradation (ERAD), and selective ER-phagy, each contribute to the maintenance of ER proteostasis (5–7). Specifically, 3 branches of the UPR (IRE1α, PERK, and ATF6) are activated in response to stress conditions within the ER to promote protein folding, while ERAD and ER-phagy are responsible for the degradative clearance of misfolded proteins (8–12) and aggregates in ER subdomains (7, 13–18). While the regulatory mechanisms of UPR and ERAD are increasingly understood (19), those of ER-phagy in vivo have lagged behind. One major challenge is to delineate how these pathways are integrated to maintain subcellular organellar networks in vivo (7). Here we report that proinsulin maturation, organellar architecture, and cell survival are synergistically impacted by ERAD and ER-phagy via IRE1α of the UPR in pancreatic β cells.

The SEL1L-HRD1 protein complex represents the most conserved component of the ERAD system, in which SEL1L functions as an obligatory cofactor for the E3 ligase HRD1 (20–23). We recently reported that loss of either β cell SEL1L-HRD1 ERAD or autophagy causes early-onset, progressive hypoinsulinemic hyperglycemia, yet remarkably through different mechanisms — triggering β cell dedifferentiation or death, respectively (5, 24). Unlike autophagy-deficient β cells, *Sel1L*-deficient β cells do not undergo detectable cell death, pointing to the existence of a possible compensatory mechanism for loss of ERAD function. Here we show, at unprecedented resolution, the intimate crosstalk among 3 ER quality-control mechanisms in the regulation of β cell function and organellar architecture in health and disease.

## Results

### Basal autophagy is activated in ERAD-deficient β cells.

**Figure 1:**
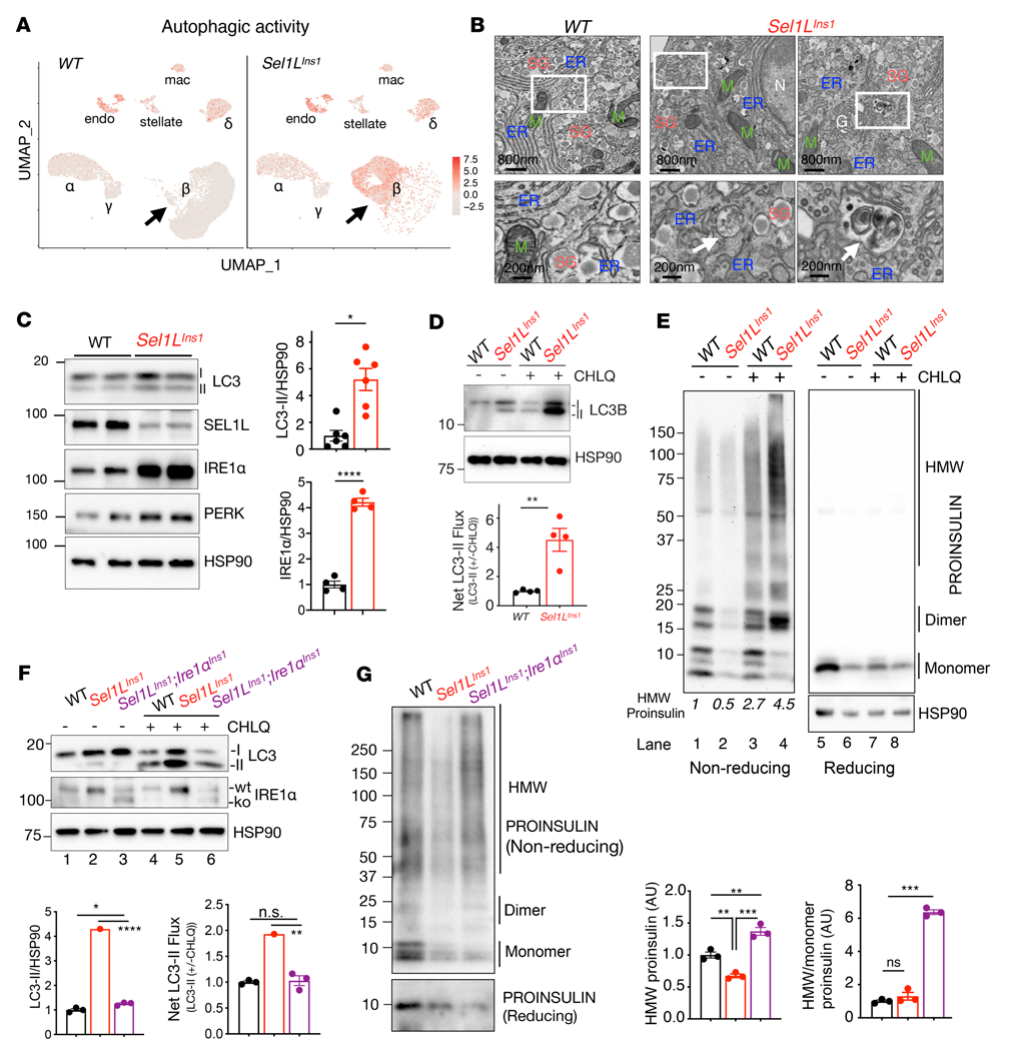
Basal autophagy is activated in ERAD-deficient β cells in part via IRE1α. (**A**) UMAP plots of islets from WT (left) and *Sel1L^Ins1^* male mice (right). Scale bars represent autophagic activity inferred from 20 reported autophagy-induced genes. (**B**) Representative TEM images of WT and *Sel1L^Ins1^* islets, showing elevated autophagosomes (arrows) in basal state (*n* = 2 mice). ER, endoplasmic reticulum; M, mitochondria; SG, secretory granules; G, Golgi. Scale bars: 800 nm (top) and 200 nm (bottom). (**C**) Western blot showing expression of autophagy and UPR genes (*n* = 4–6 per group). (**D**) Western blot showing expression of LC3 in the presence of chloroquine (CHLQ) for 2 hours, with quantitation of net LC3 flux shown below (*n* = 4). (**E**) Western blot analyses, under nonreducing (without dithiothreitol [–]) and reducing (+) conditions, of proinsulin in islets with or without treatment with chloroquine (2 independent repeats). (**F**) Western blot showing basal autophagy and autophagic flux in islets from indicated genotypes (quantitation is shown below, *n* = 3). (**G**) Western blotting analyses, under nonreducing and reducing conditions, of proinsulin in islets of indicated genotypes (*n* = 3), with quantification shown on the right. Values are shown as mean ± SEM. *P < 0.05; **P < 0.005; ***P < 0.001; ****P < 0.0001 by unpaired, 2-tailed Student’s *t* test (**C** and **D**) or 1-way ANOVA with Tukey’s post hoc test (**F** and **G**).

Single-cell sequencing analyses of *Sel1L^Ins1^* islets, in which *Sel1L* is knocked out using a Cre driver under control of the β cell–specific endogenous *Ins1* promoter (24), revealed that, in addition to ER processing pathways (24), genes involved in autophagy were enriched in *Sel1L^Ins1^* β cells (Supplemental Figure 1A; supplemental material available online with this article; https://doi.org/10.1172/JCI163584DS1). This was further confirmed using a curated list of autophagy-induced genes, defined by Bordi et al. (25), to infer “autophagic activity” (Figure 1A and Supplemental Figure 1, B and C). Transmission electron microscopy (TEM) analysis revealed increased autophagosomes in *Sel1L^Ins1^* islets (Figure 1B, arrows), which were undetectable in control (WT) β cells under basal conditions, as previously reported (26). Autophagy substrate p62 protein was decreased, albeit not statistically significant, in *Sel1L^Ins1^* islets (data not shown), while the Atg7-dependent conversion of microtubule-associated protein 1 light chain I (LC3-I) to LC3-II (LC3-phosphatidylethanolamine conjugate) was significantly elevated in *Sel1L^Ins1^* islets compared with those in WT islets (Figure 1C). Treatment with chloroquine, an inhibitor of autophagosome-lysosome fusion (27), elevated LC3-II levels significantly more in *Sel1L^Ins1^* islets than that of WT islets, indicative of increased autophagic flux (Figure 1D). Consistent with elevated autophagy, high molecular weight (HMW) proinsulin conformers (a known substrate of autophagy; refs. 28, 29) were reduced in *Sel1L^Ins1^* islets compared with WT islets (Figure 1E; lane 1 vs. 2). Acute blockade of autophagy using chloroquine, however, greatly enhanced the abundance of proinsulin dimers and HMW conformers, formed via disulfide bonds (thus sensitive to reducing agent) in *Sel1L^Ins1^* islets (Figure 1E, lane 4 vs. 2).

### Increased autophagic activity in Sel1L^Ins1^ islets is mediated, in part, by IRE1α.

IRE1α is an endogenous substrate of SEL1L-HRD1 ERAD (19, 30) and previous studies have shown that the IRE1α branch of the UPR can activate autophagy in vitro (31, 32). We next asked whether IRE1α links ERAD to autophagy activation in β cells. In line with observations in other Sel1L-deficient cell types (19, 30), IRE1α protein level was elevated approximately 4-fold in *Sel1L^Ins1^* islets, but UPR sensor PERK was unchanged (Figure 1C). To determine whether IRE1α enhances autophagy in *Sel1L^Ins1^* islets, we generated β cell–specific *Sel1L*;*Ire1a* double-knockout (*Sel1L^Ins1^;Ire1a^Ins1^*) mice. Surprisingly, deletion of the RNase domain of IRE1α in *Sel1L^Ins1^* islets reduced LC3-II to levels comparable to that of WT islets (Figure 1F, lane 3 vs. 2 and 6 vs. 5). It should be noted that the autophagic activity was not completely blocked by deletion of *Ire1a*, as treatment with chloroquine still increased LC3-II flux in *Sel1L^Ins1^;Ire1a^Ins1^* islets (Figure 1F, lane 3 vs. 6). Moreover, deletion of IRE1α significantly elevated HMW conformers of proinsulin in *Sel1L^Ins1^* islets (Figure 1G). Taken together, these data demonstrated that SEL1L-HRD1 ERAD deficiency in β cells triggers the activation of basal autophagy toward proinsulin aggregates, at least in part, via IRE1α.

### Synergism of ERAD and autophagy in β cell survival and systemic glucose homeostasis.

**Figure 2:**
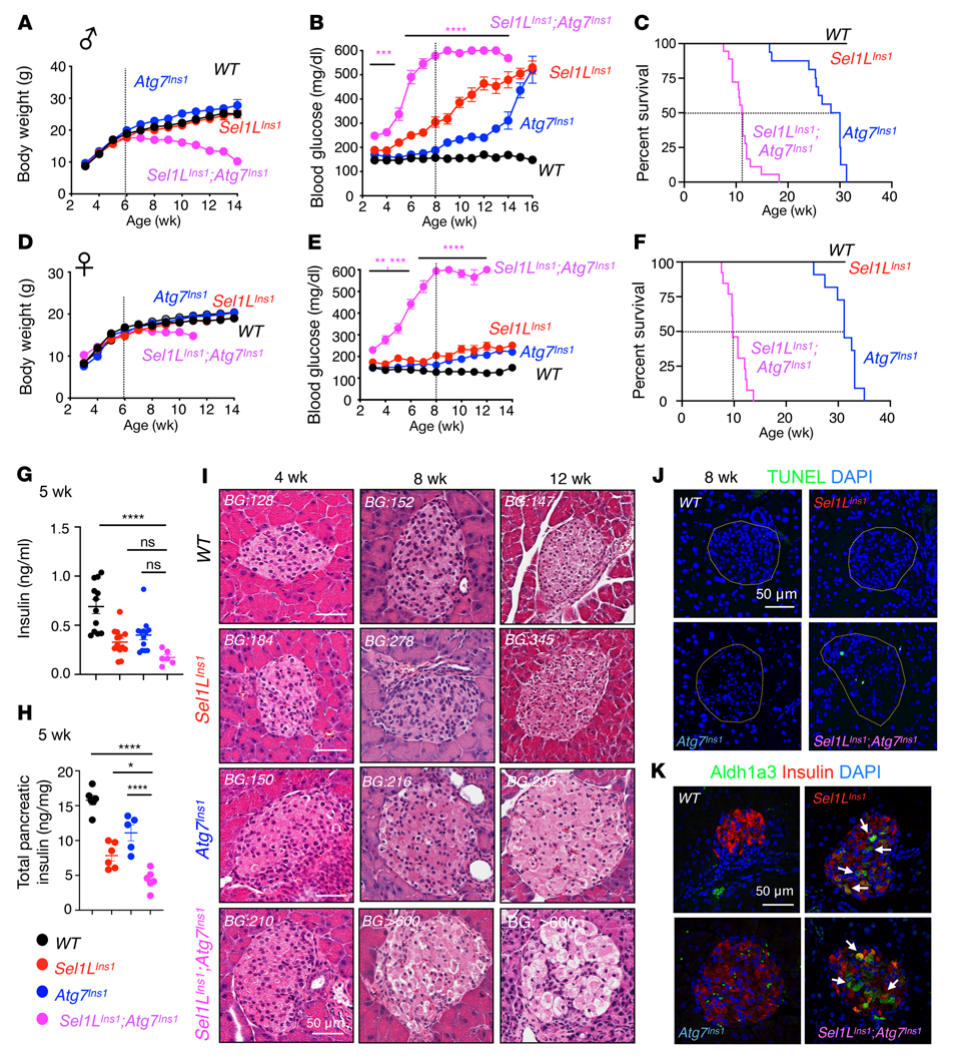
Synergistic effect of β cell ERAD and autophagy on β cell and organismal survival and systemic glucose homeostasis. (**A**–**F**) Growth curves (**A** and **D**, *n* = 6–10 and 6–12 per group), ad libitum blood glucose (**B** and **E**, *n* = 6–10 and 6–12 per group), and survival curves (**C** and **F**, *n* = 16–20 and 8–12 per group) of male (**A**–**C**) and female littermates (**D**–**F**). Error bars indicate SEM. **P < 0.005, ***P < 0.001, ****P < 0.0001 by mixed-effect analysis with Tukey’s post hoc test. In panel **E**, **P < 0.005 for the 4-, 5-, and 6-week time points, and ***P < 0.001 for the 3-week time point. (**G**) Serum insulin levels (male and female combined, *n* = 6–12 per group) and (**H**) total pancreatic insulin content (male, *n* = 5–6 per group) at 5 weeks. Values are shown as mean ± SEM. *P < 0.05, ****P < 0.0001 by 1-way ANOVA followed by Tukey’s post hoc test. (**I**) H&E images of paraffin-embedded pancreas sections from different genotypes at 4, 8, and 12 weeks. BG, ad libitum blood glucose. (**J**) Representative TUNEL staining at 8 weeks (quantitation shown in Supplemental Figure 3A). (**K**) Representative Aldh1a3 staining at 8 weeks. Scale bars: 50 μm.

To further test the hypothesis that compensatory upregulation of autophagy may limit proinsulin aggregation and cell death in *Sel1L^Ins1^* islets, we generated *Sel1L*- and *Atg7*-deficient (*Sel1L^Ins1^*;*Atg7^Ins1^* [DKO]) mice. *Sel1L^fl/fl^*;*Atg7^fl/fl^* (WT) and single KO (*Sel1L^Ins1^* and *Atg7^Ins1^*) mice were included as controls. Initial growth within the first 6 weeks of life was comparable among the 4 cohorts, but DKO male mice started to lose body weight around 7–8 weeks of age (Figure 2A). Tissue histology revealed no abnormalities in peripheral white and brown adipose tissues or the liver of 8-week-old mice (Supplemental Figure 2, A–C). Ad libitum blood glucose levels were slightly increased in DKO male mice at weaning, but quickly rose to over 600 mg/dL (glucometer detection limit) around 8 weeks of age (Figure 2B). The DKO mice died prematurely, with a median life span of 11 weeks (Figure 2C). By contrast, the hyperglycemia of *Sel1L^Ins1^* and *Atg7^Ins1^* littermates initiated around 5 and 8 weeks of age, respectively, and progressed at a much slower pace over the next 10 weeks (Figure 2B). Median survival was approximately 30 weeks for *Atg7^Ins1^* mice, while *Sel1L^Ins1^* littermates lived longer (Figure 2C). Similar observations were obtained in female cohorts (Figure 2, D–F) with regard to body weight, blood glucose, and survival rate. In line with our previous report (24), blood glucose increased more modestly in single KO female cohorts compared with the males. At 5 weeks of age, both serum insulin and total pancreatic insulin in DKO mice were reduced to about half of those in *Sel1L^Ins1^* and *Atg7^Ins1^* littermates and a third of those in WT mice (Figure 2, G and H).

Histological examination of islets revealed that DKO islets deteriorated much faster than either of the single KO mice (Figure 2I). At 8 weeks of age, vacuolization and cell death (i.e., TUNEL-positive cells) were pervasive in DKO β cells, but not in single KO cohorts (Figure 2, I and J, and quantitated in Supplemental Figure 3A). In line with our recent study (24), β cell dedifferentiation as marked by elevated Aldh1a3 expression was comparable in *Sel1L^Ins1^* islets with or without Atg7, but was undetectable in WT or *Atg7^Ins1^* islets (arrows, Figure 2K). Consistently, expression of the β cell identity marker MafA was reduced in both *Sel1L^Ins1^* and DKO islets (arrows, Supplemental Figure 3B), with centrally located glucagon-positive α cells (arrows, Supplemental Figure 3C). Taken together, these data demonstrated that SEL1L-HRD1 ERAD and autophagy play a synergistic role in β cell survival, systemic glucose homeostasis, and organismal survival. Indeed, autophagy activation in *Sel1L^Ins1^* islets prevents β cell death.

### Synergism of ERAD and autophagy in ER maturation of nascent proinsulin.

**Figure 3:**
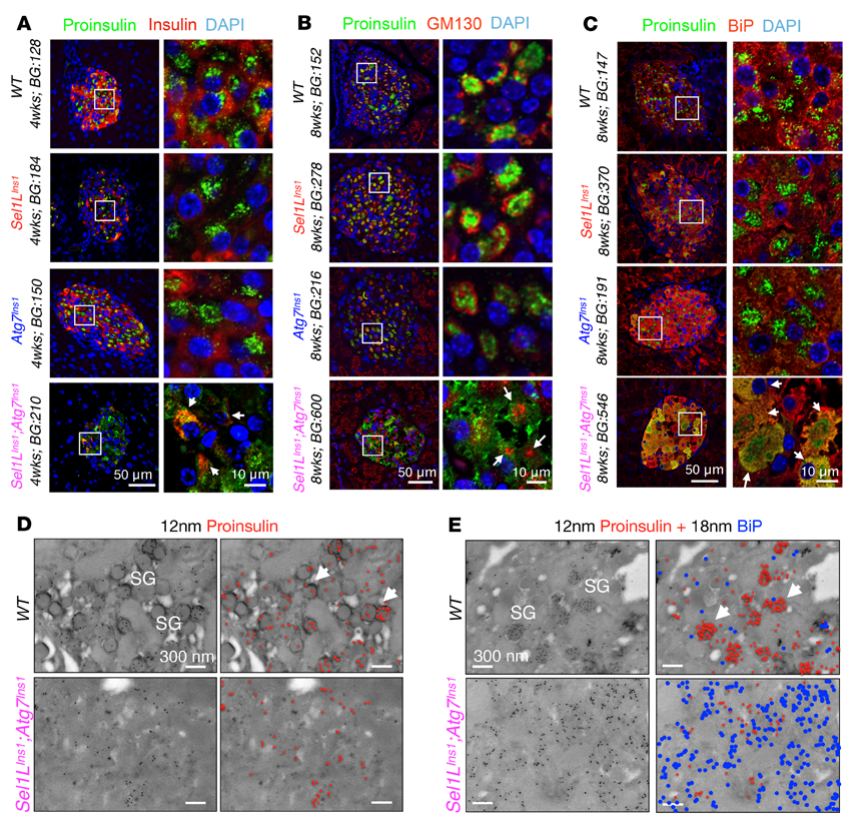
ERAD and ER-phagy synergistically ensure the ER exit of nascent proinsulin in β cells. (**A**–**C**) Representative confocal images of (**A**) proinsulin and insulin, (**B**) GM130 and proinsulin, and (**C**) BiP and proinsulin (*n* = 3 mice per genotype in each group). Scale bars: 50 μm (left columns) and 10 μm (right columns). BG, blood glucose (mg/dL). (**D** and **E**) Representative TEM following immunogold labeling of proinsulin (12 nm gold) (**D**), and proinsulin and BiP (18 nm gold) (**E**) in primary islets. White arrows in WT islets indicate secretory granules. Color-coded gold particles are shown on the right (proinsulin in red and BiP in blue). Scale bars: 300 nm. SG, secretory granule.

We next investigated the maturation of nascent proinsulin in the ER in 4- to 8-week-old mice. While the rate of proinsulin biosynthesis in islets was comparable among the cohorts (Supplemental Figure 4A), there was very little mature insulin in DKO islets (Figure 3A). Moreover, unlike in WT and single KO islets where proinsulin predominantly localized as a juxtanuclear cluster adjacent to GM130 (the *trans*-Golgi compartment; Figure 3B), proinsulin was largely retained in the ER (colocalized with ER chaperone BiP; Figure 3C and Supplemental Figure 4C) and failed to reach the *trans*-Golgi region in DKO islets (Figure 3, B and C). Immunogold labeling of proinsulin (specific for β cells, not α cells; Supplemental Figure 4B) showed that, unlike in WT β cells where proinsulin was predominantly found in clusters of nascent secretory granules devoid of BiP (arrows; Figure 3, D and E), proinsulin seemed to distribute diffusely and become colocalized with BiP in DKO islets (Figure 3, D and E). Hence, SEL1L-HRD1 ERAD and autophagy play a synergistic role in the maturation and ER exit of proinsulin.

### ER-phagy is activated in Sel1L^Ins1^ islets.

**Figure 4:**
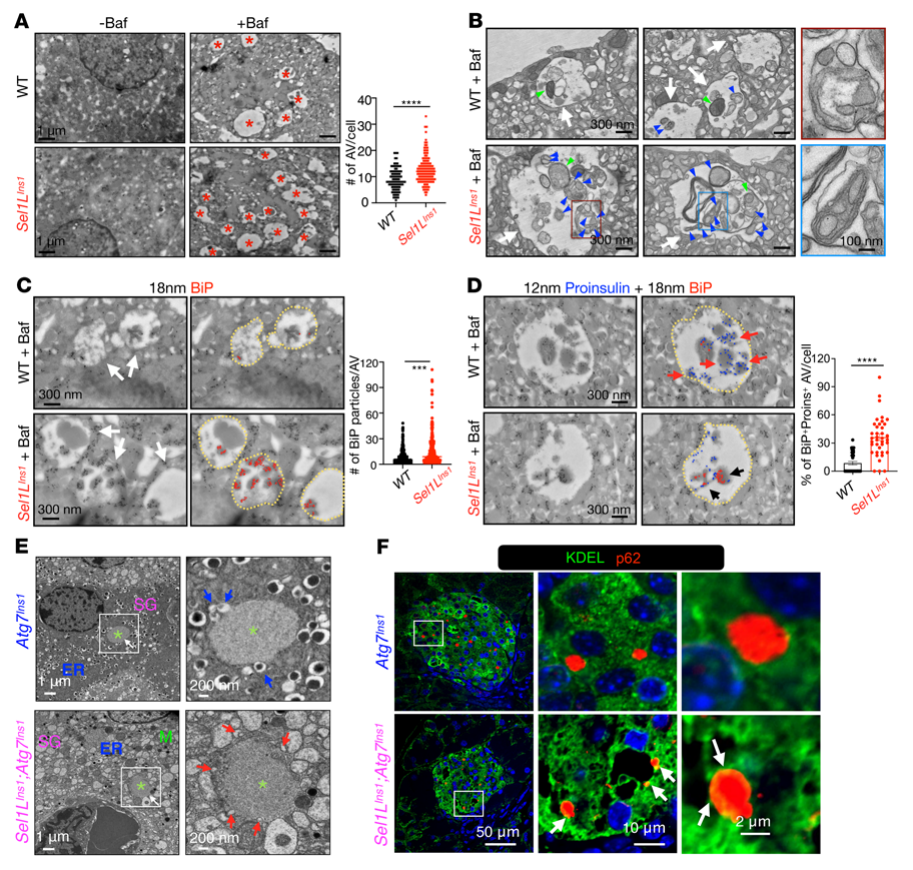
ERAD deficiency enhances ER-phagy of ER-retained proinsulin in β cells. (**A**) Representative TEM images showing autophagic vacuoles (AV), marked by red asterisk (right panel) in bafilomycin-treated (Baf, 100 nM for 2 hours) islets of WT and *Sel1L^Ins1^* mice. Quantitation of AVs per cell is shown on the right (*n* = 100–130 β cells from 2 mice for each genotype). Scale bars: 1 μm. (**B**) Zoomed TEM images showing ER-like structures (blue arrows) and mitochondria (green arrows) in AVs (white arrows). Boxed area shows magnified images highlighting the ER inside the AVs. Scale bars: 300 nm and 100 nm (zoomed images on right). (**C**) Immunogold labeling against BiP (diameter, 18 nm), AVs are marked by yellow circles and gold particles are color coded in the right panel. Quantitation of total BiP particles in each AV is shown on the right; *n* = 50–100 β cells from 2 mice for each genotype. (**D**) Immunogold labeling against BiP (diameter, 18 nm) and proinsulin (diameter, 12 nm); gold particles are color coded in the right panel. Quantitation of percentage of BiP^+^Proins^+^ AV per cell is shown on the right (*n* = 40–50 β cells from 2 mice for each genotype. Scale bars: 300 nm. ***P < 0.001, ****P < 0.0001 by unpaired, 2-tailed Student’s *t* test. (**E**) TEM of pancreatic islets from *Atg7^Ins1^* and *Sel1L^Ins1^;Atg7^Ins1^* mice. Asterisks indicate aggregates; secretory granules (SG) are marked by blue arrows and ER by red arrows. Scale bars: 1 μm (left) and 200 nm (right). (**F**) Representative confocal images of KDEL and p62 staining in pancreatic islets from *Atg7^Ins1^* and *Sel1L^Ins1^;Atg7^Ins1^* mice. Arrows indicate colocalization of KDEL and p62 signals. Scale bars: 50, 10, and 2 μm (left to right).

Accumulation of proinsulin in the ER of DKO islets suggested a possible role of ER-phagy in the clearance of proinsulin in ERAD-deficient islet. We next asked how ERAD and autophagy play a synergistic role in β cells. To visualize autophagy and ER-phagy in β cells, we performed TEM of primary islets acutely treated with bafilomycin, a drug that causes the accumulation of autophagic vacuoles (AVs), i.e., autophagolysosomal intermediates (18). Consistent with increased autophagic flux, bafilomycin treatment revealed that there were significantly more AVs in *Sel1L^Ins1^* β cells than WT β cells (asterisks, Figure 4A). Both mitochondria (green arrows) and ribosome-studded ER-like fragments (blue arrows) could be found in the AVs (Figure 4B). More importantly, these ER-like fragments (blue arrowheads) were more abundant in AVs of *Sel1L^Ins1^* β cells than those in WT β cells. Immuno-EM labeling of BiP, a luminal ER marker, demonstrated that these structures are indeed the ER and they were significantly more abundant in *Sel1L^Ins1^* β cells than those in WT β cells (Figure 4C). Furthermore, colabeling of proinsulin and BiP followed by TEM revealed that proinsulin was detected in the AVs but was mostly BiP free in WT β cells (red arrows, Figure 4D), consistent with the notion that autophagy degrades proinsulin in the secretory granules of WT β cells (28, 29). By contrast, proinsulin in the AVs of *Sel1L^Ins1^* β cells was largely associated with BiP (black arrows, Figure 4D), indicative of ER-phagy of ER-retained proinsulin in *Sel1L^Ins1^* β cells.

To determine whether ER-phagy observed in ERAD-deficient islets is specific to misfolded proinsulin or a general mechanism for ER recycling, we transfected WT and *HRD1^–/–^* HEK293T cells with an ER-phagy reporter, composed of tandem monomeric RFP and GFP sequences flanked by a signaling sequence and the ER retention signal KDEL at the N- and C-terminus (33)(Supplemental Figure 5A). Following ER-phagy, RFP would be cleaved from the reporter, which can be detected by Western blotting. Indeed, our data showed that the cleaved RFP protein level was over 3 times higher in *HRD1^–/–^* HEK293T cells compared with that in WT HEK293T cells (Supplemental Figure 5B), indicative of ER-phagy activation in cells with impaired SEL1L-HRD1 ERAD function. Providing further support for the role of ER-phagy in *Sel1L^Ins1^* islets, TEM analysis revealed that p62-positive foci (green asterisks) were surrounded by insulin granules (blue arrows) and ER tubules (red arrows) in *Atg7^Ins1^* and DKO β cells, respectively (Figure 4E). Moreover, p62 partially colocalized with KDEL in DKO β cells, to a much more significant extent than that in *Atg7^Ins1^* β cells (Figure 4F and Supplemental Figure 5C). Taken together, our data showed that SEL1L-HRD1 ERAD deficiency in β cells enhances ER-phagy–mediated clearance of ER.

### RTN3-mediated ER-phagy of misfolded proinsulin in Sel1L^Ins1^ islets.

ER-phagy is mediated by ER-phagy adaptors, a family of ER membrane proteins with LC3 binding regions, including (but not limited to) reticulon-3 (RTN3), FAM134, CCPG1, TEX264, and SEC62 (34). As misfolded mutant proinsulin AKITA is known to be selectively degraded in an RTN3-dependent manner (35, 36), we next tested whether WT proinsulin is misfolded and forms aggregates in ERAD-deficient cells, which can be targeted by RTN3. Indeed, in HEK293T cells lacking both ERAD and RTN3, there was a marked increase in HMW proinsulin isoforms compared with those lacking either ERAD or RTN3 (Supplemental Figure 5E, lanes 5 and 6 vs. lanes 1–4, and Supplemental Figure 5D). Furthermore, immunofluorescent staining revealed that proinsulin appeared in large punctae colocalized with BiP in COS7 cells lacking both HRD1 and RTN3 (Supplemental Figure 5F). In contrast, loss of another ER-phagy adaptor, FAM134, did not induce proinsulin aggregation in ERAD-deficient cells (Supplemental Figure 5, G and H). Taken together, our data suggested that RTN3 may play a role in the clearance of HMW proinsulin aggregates by ER-phagy in the absence of SEL1L-HRD1 ERAD.

### ERAD and autophagy reshape organellar network in β cells.

**Figure 5:**
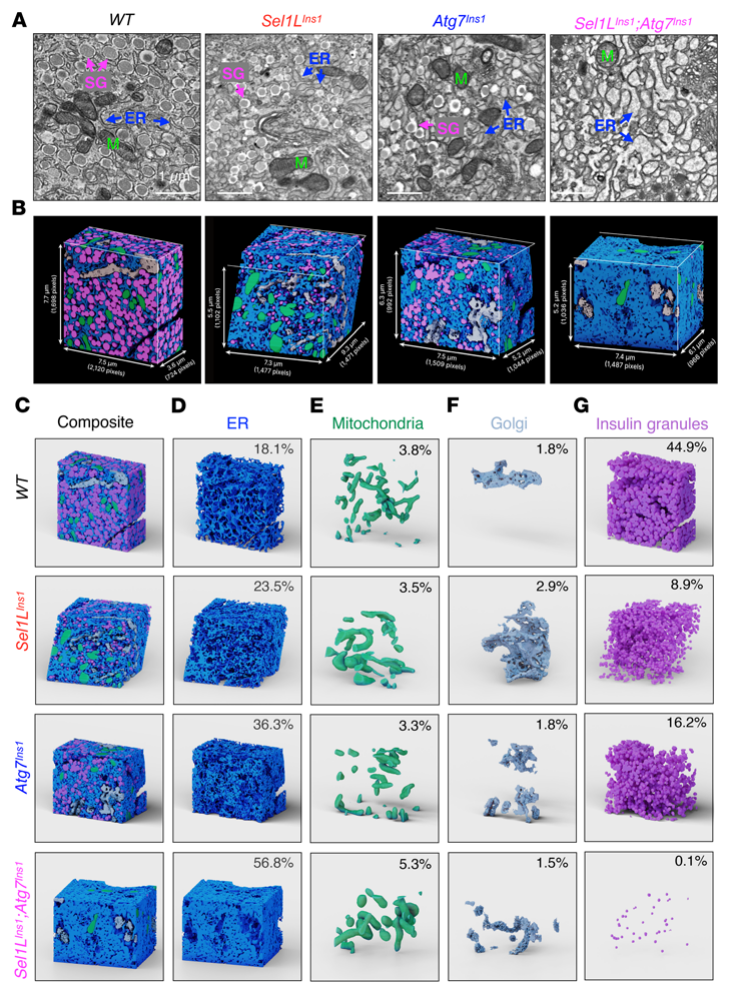
ERAD and ER-phagy synergistically reshape the organellar network in β cells. (**A**) TEM of pancreatic islets from 8-week-old male WT, *Sel1L^Ins1^*, *Atg7^Ins1^*, and *Sel1L^Ins1^;Atg7^Ins1^* mice. Scale bars: 1 μm. Arrows indicate ER; M, mitochondria; SG, secretory granules. (**B**) 3D reconstruction of FIB-SEM images using convolutional neural network–based automated segmentation of organelles in β cell. Dimensions of each volume are indicated. Blue, ER; green, mitochondria; white, Golgi; pink, insulin granules. (**C**–**G**) 3D reconstruction of FIB-SEM images showing ER (**D**), mitochondria (**E**), Golgi (**F**), and insulin granules (**G**) with their respective volume expressed as percentages of reconstructed cell volume.

To directly visualize the organellar network, we next performed TEM of islets from the 4 genotypes (Supplemental Figure 6A). WT β cells had a typical architecture of cytoplasm filled with insulin granules in close proximity to slender ER sheets (arrows) (Figure 5A). The β cells in both *Sel1L^Ins1^* and *Atg7^Ins1^* islets had fewer insulin granules and slightly dilated ER compared with WT β cells. On the other hand, DKO β cells were almost completely devoid of insulin granules, but rather filled with dense ER networks in the cytoplasm (Figure 5A). These massive changes were limited to β cells, as non-β endocrine cells in DKO islets appeared entirely normal compared to those in WT islets (Supplemental Figure 6B).

We then utilized focused ion beam scanning electron microscopy (FIB-SEM) imaging (37) of the islets from 8-week-old mice for a detailed 3D evaluation (Supplemental Figure 7A). We imaged a volume of β cells at a voxel size of approximately 5 nm in *x*, *y*, and *z* dimensions for each genotype. The volume and slice information are detailed in the Methods section. Using machine learning–based approaches and convolution neuronal networks, we next generated ground truth for the ER, mitochondria, Golgi, and insulin granules (Supplemental Figure 7B), which were further fine-tuned until a good quality of segmentation was reached for automatic segmentation in 3D (Figure 5B). We next calculated the percentage volume for the individual organelles and noted that the abundance of the ER (per total cell volume) increased from 18% in WT to 23% and 36% in *Sel1L^Ins1^* and *Atg7^Ins1^* β cells, reaching 57% in DKO β cells (Figure 5, C and D). On the other hand, the abundance of insulin granules (per total cell volume) dropped from 44% in WT, to 9%, 16%, and 0.1% in *Sel1L^Ins1^*, *Atg7^Ins1^*, and DKO β cells, respectively, while the percentage of mitochondria and Golgi was comparable among the 4 genotypes (Figure 5, E–G, and Supplemental Figure 7C). Of note, individual mitochondria appeared enlarged in *Sel1L^Ins1^* and DKO β cells compared with those in WT and *Atg7^Ins1^* β cells (Figure 5E and quantitated in Supplemental Figure 7D), in line with our recent findings in cold-stimulated brown adipocytes (37).

### ERAD and autophagy synergistically determine the ER architecture of β cells.

**Figure 6:**
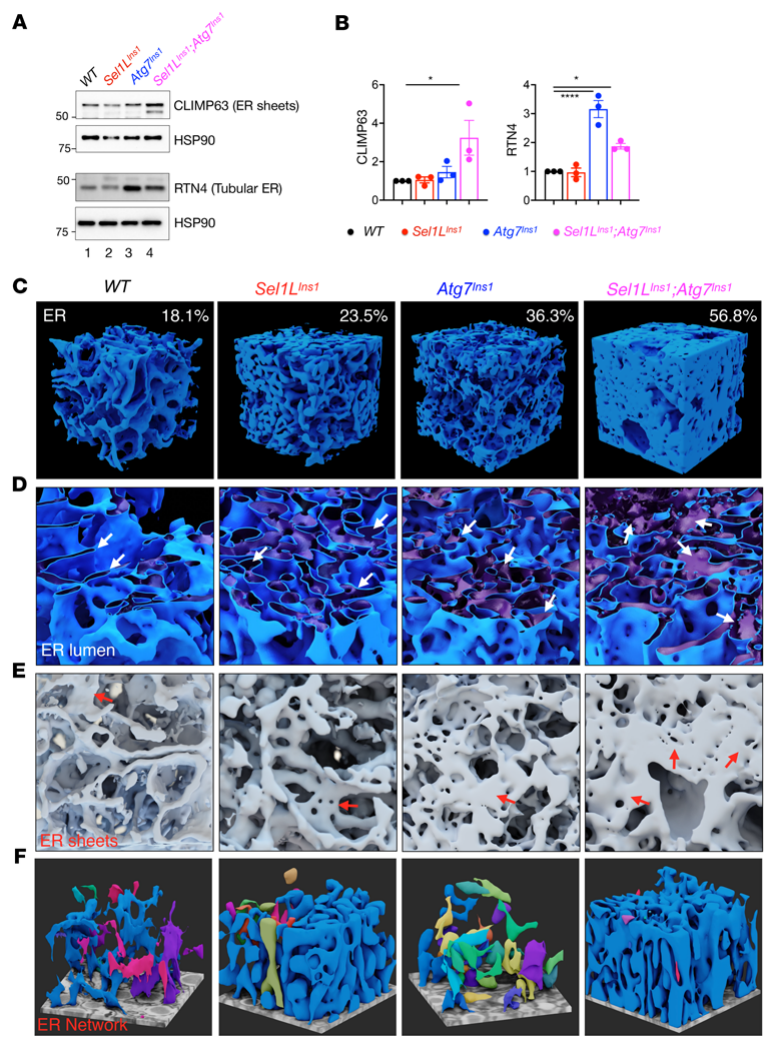
ERAD and autophagy synergistically determine the ER architecture in β cells. (**A**) Immunoblot of ER sheet and tubule markers. (**B**) Quantification of results in **A**; *n* = 3 mice per genotype. Values are shown as mean ± SEM. *P < 0.05, ****P < 0.0001 by 1-way ANOVA with Tukey’s post hoc test. (**C**) Magnified view of the ER (3 μm × 3 μm × 3 μm cubic volume) in each genotype. (**D**) Magnified view of the of the ER (*x*-*y* plane), highlighting the size of ER lumen. (**E**) Magnified view of the ER (*y*-*z* plane). Arrow indicates ER sheets. (**F**) 3D reconstruction of the fraction of ER lumen highlighting ER networks (2 μm × 2 μm × 1 μm), with individual ER networks in distinct colors.

Lastly, we explored in greater detail how ERAD and autophagy machineries alter ER architecture. The ER is a highly dynamic organelle that remodels between a reticular network of tubules and flattened sheets, which are important not only for interorganellar communication, but also degradation of misfolded proteins (36). Both sheet (CLIMP63) and tubular (RTN4) proteins were significantly higher in DKO islets compared with those of WT islets (Figure 6A, lane 4 and quantitated in Figure 6B), pointing to an expansion of both sheet and tubular structures of the ER. On the other hand, in *Atg7^Ins1^* islets, tubular marker RTN4, but not CLIMP63, was highly elevated (Figure 6A, lane 3, and quantitated in Figure 6B), indicative of the expansion of mostly tubular ER. *Sel1L^Ins1^* islets, on the other hand, had both sheet and tubular ER marker levels comparable to those of WT islets (Figure 6A, lanes 1 and 2, and quantitated in Figure 6B).

We next extracted an equal volume of ER (3 μm × 3 μm × 3 μm) from all genotypes in FIB-SEM analysis to better visualize the change in ER morphology at the 3D level. Strikingly, a combined loss of ERAD and autophagy in β cells not only triggered a massive expansion of the ER (Figure 6C), but also the expansion of the ER luminal volume (arrows, Figure 6D). The ER lost its sheet-like morphology, but rather exhibited fenestrated sheet structures in DKO β cells (Figure 6E). Restricted segmentation of the ER lumen further revealed a distinct spatial organization of the ER network in each genotype (individual ER networks in distinct colors, Figure 6F and Supplemental Videos 1–4). While the ER exhibited as thin sheets with less extensive interconnections in WT and *Atg7^Ins1^* β cells, loss of Sel1L in *Sel1L^Ins1^* β cells led to the dilation of ER lumen and the formation of much denser ER network, and to a much more extensive extent in DKO β cells (Figure 6F and Supplemental Videos 1–4). Taken together, these findings suggested that ERAD and ER-phagy together have a profound and synergistic effect on the ER architecture and network in β cells.

## Discussion

Pancreatic β cells synthesize and fold thousands of proinsulin molecules per minute in the ER before being stored in the secretory granules after proteolytic processing in the endosomes (1, 2); hence, these cells exhibit unique spatial organization of the organelles (4). These organelles may undergo rapid reshaping in response to changes in the cellular environment and energy/nutrient status. A major challenge is to visualize these subcellular architectures in 3D in their native environment and delineate the mechanism(s) underlying the remodeling. Furthermore, while there is a growing recognition that ER homeostasis in β cells is important for proinsulin biogenesis (1), our understanding of the importance of ER-phagy and its regulation, as well as the integration of various quality control pathways in vivo, remains very limited.

Here, using various genetic KO mouse models powered by high-resolution 2D and 3D imaging techniques, our data demonstrate an intricate crosstalk between SEL1L-HRD1 ERAD and autophagy/ER-phagy, in part linked by IRE1α of the UPR, to limit the accumulation of misfolded proinsulin in the ER and thus to promote ER health/homeostasis and cellular function. The crosstalk between SEL1L-HRD1 ERAD and ER-phagy ensures the folding and maturation of nascent proinsulin in the ER of β cells by clearing misfolded proinsulin protein and aggregates, respectively, thereby providing a conducive environment for nascent protein biogenesis. It is important to note that, while many recent studies showed the importance of ER-phagy in the clearance of particular misfolded substrates in vitro and in vivo (6, 7, 38, 39), our data show that SEL1L-HRD1 ERAD activity in β cells limits the activity of ER-phagy under basal conditions. In other words, our data demonstrate that SEL1L-HRD1 ERAD function indeed represses ER-phagy activity, presumably by preventing the formation of ER aggregates (from misfolded proteins) and/or limiting the total abundance of IRE1α (Supplemental Figure 8A). Indeed, upregulation of ER-phagy in the face of ERAD dysfunction appears to be an adaptive mechanism for cells to survive the accumulation of misfolded ER proteins (Supplemental Figure 8B). The synergy between ERAD and autophagy in β cells is clearly demonstrated by the synthetic lethality in the DKO mice (Figure 2) and by the profound alteration in proinsulin maturation and organellar network (Figures 3–6). While our study suggests the possible role of IRE1α-XBP1–mediated upregulation of autophagic activity as previously reported (32, 40), future studies are required to delineate mechanistically how IRE1α links the ERAD and autophagy in β cells and the extent to which other branches of the UPR such as the PERK and ATF6 pathways may contribute.

In yeast and mammalian cells in vitro, it has been reported that induction of ER stress alters ER morphology and increases ER volume (41), while ER-phagy trims excess ER membrane in response to ER stress (42, 43). Nonetheless, how ER quality-control mechanisms work cooperatively on ER architecture and remodeling has previously been unexplored. Our data provide unprecedented resolution of the ER network architecture at a 3D level in β cells, demonstrating that SEL1L-HRD1 ERAD and autophagy are 2 key regulators of ER architecture and volume in β cells (Supplemental Figure 8C). Deletion of *Sel1L* triggers dilation and mild expansion of the ER, whereas loss of *Atg7* results in increased tubular and fragmented ER in β cells (Figure 6). Simultaneous loss of both SEL1L-HRD1 ERAD and autophagy leads to massive expansion of both ER lumen volume and the ER network, including both ER tubules and sheets (Supplemental Figure 8C). Similar to a recent study on hepatocytes (44), future studies are required to explore how the ER remodels in vivo. Nonetheless, this study not only delineates the pathophysiological importance of a synergistic action of SEL1L-HRD1 ERAD and autophagy/ER-phagy in β cell biology, but also provides a framework for understanding how β cell quality-control pathways contribute to ER homeostasis in the pathogenesis and treatment of diabetes.

## Methods

### Mice.

*Sel1L^fl/fl^* (20), *Atg7^fl/fl^* (45), and β cell–specific *Sel1L^Ins1^* and *Atg7^Ins1^* mice on the C57BL/6J background were generated as we recently reported (24). *Ire1a^fl/fl^* mice were obtained from Takao Iwawaki (46), as we recently reported (30). Activation of Cre results in deletion of exons 20 and 21 from the floxed *Ire1a* allele, resulting in IRE1α RNase–deficient mice. Double-knockout mice (*Sel1L^Ins1^;Atg7^Ins1^* and *Sel1L^Ins1^;Ire1a^Ins1^*) were generated by initially crossing the double-floxed mice with *Ins1*-*Cre* mice. All mice were housed in an ambient temperature room with 12-hour light cycle and fed a low-fat diet (13% fat, 57% carbohydrate, and 30% protein, LabDiet 5LOD). Body weight and blood glucose were monitored weekly in all genotypes. Some genotypes developed severe high blood glucose accompanied by swollen abdomen and hunched back with age. When these mice appeared scruffy and hunched with overall poor health, they had to be euthanized and thus pronounced dead (for analysis of survival curve). Both male and female cohorts were analyzed, and no sex-specific differences were observed.

### Serum metabolite analysis.

Serum levels of insulin were measured using an ultrasensitive ELISA (Crystal Chem) as per the manufacturer’s instructions.

### Islet isolation.

Pancreatic islets were isolated from mice as previously described (24, 47). Briefly, mice were sacrificed by cervical dislocation and immediately processed for pancreatic perfusion. The pancreas was distended via the intraductal injection of Liberase (Roche, 5401020001) and incubated at 37°C for digestion. The digested suspension was passed through a nylon mesh and islets were isolated by density gradient centrifugation on a Histopaque gradient (1.077 g/mL density; Sigma-Aldrich) for 20 minutes at 900*g* without brake. Islets were then collected from the interface, washed, and hand-picked under a dissecting microscope. Isolated islets were recovered overnight in RPMI 1640 medium in a humidified incubator (95% air, 5% CO_2_) at 37°C.

### Pancreatic insulin content.

Pancreases were isolated, weighed, placed into 3 mL of acid-ethanol solution (1.5% HCl in 75% [v/v] ethanol in water), and homogenized for 30 seconds. The homogenate was rotated for 24 hours at 4°C for insulin extraction. After centrifugation at 1800*g* for 30 minutes at 4°C, supernatant was diluted, and insulin content was measured as above.

### TEM.

The pancreas was quickly removed from the mice, chopped into tiny pieces (approximately 1–2 mm cubes), and fixed in 2.5% glutaraldehyde and 4% paraformaldehyde in 0.1 M Na-cacodylate buffer for 1 hour. Thereafter, the samples were submitted to the University of Michigan Microscopy Core for washing, embedding, sectioning, and imaging via TEM.

### FIB-SEM segmentation.

The organelles were segmented using the 3dEMtrace platform of ariadne.ai (https://ariadne.ai/). In brief, ground truth data were generated from the raw FIB-SEM images by manually annotating organelles. Deep convolutional neural networks were trained using the manually generated ground truth and fine-tuned until a good quality of segmentation was reached for automated segmentation in 3D. The segmentations were then proofread and cleaned up by expert inspection using the open-source software Knossos (https://knossos.app/). For each segmentation class, meshes and binary Tiff masks were generated for subsequent rendering and visualization in Knossos and in the open-source 3D visualization tool Blender (https://www.blender.org/).

### Statistics.

Results are expressed as the mean ± SEM unless otherwise stated. Statistical analyses were performed in Prism (GraphPad Software Inc.). Comparisons between 2 groups were made by unpaired, 2-tailed Student’s *t* test. One-way ANOVA followed by Tukey’s post hoc test was used to determine statistical significance for more than 2 groups. A *P* value of less than 0.05 was considered statistically significant. All experiments were repeated at least twice, or performed with several independent biological samples, and representative data are shown.

### Study approval.

All animal procedures were approved by and done in accordance with the IACUC at the University of Michigan Medical School (PRO00010658).

## Author contributions

NS designed and performed most of experiments. MT performed the TEM and FIB-SEM. JZ performed some staining and assisted with data analysis. YL analyzed the single-cell sequencing data. LH performed the pulse-chase experiments. RBR provided insightful discussions. JK and JC performed some in vitro experiments. GP and APA assisted in the FIB-SEM data analysis. PA and BT contributed to experimental design and discussions. LQ designed and supervised the project. LQ and NS wrote the manuscript, and all other authors edited and approved the manuscript.

## Supplementary Material

Supplemental data

Supplemental video 1

Supplemental video 2

Supplemental video 3

Supplemental video 4

## Version 1 11/08/2022

In-Press Preview

## Version 2 01/03/2023

Electronic publication
