# Supplementary material for: Integration of ER protein quality control mechanisms defines β cell function and ER architecture
Source: J Clin Invest. 2023 Jan 3;133(1):e163584. doi: 10.1172/JCI163584 (PMC9797341; doi:10.1172/JCI163584)
Supplement: Supplemental data [file jci-133-163584-s034.pdf]

Supplementary Materials for

Integration of ER Protein Quality Control Mechanisms Defines β-Cell Function and ER Architecture

Neha Shrestha[1,2], Mauricio Torres[1], Jason Zhang[3], You Lu[1], Leena Haataja[2], Rachel B. Reinert[2], Jeffrey Knupp[4], Yu-Jie Chen[4], Gunes Parlakgul[5], Ana Paula Arruda[5,6], Billy Tsai[4], Peter Arvan[1,2], Ling Qi[1,2]*

MATERIALS AND METHODS

Hematoxylin and eosin staining (H&E) and morphometric analyses. Pancreas were isolated, fixed in 10% neutral buffered formalin (VWR 95042-908) overnight at 4°C and processed by the University of Michigan Comprehensive Cancer Center for paraffin embedding, sectioning and H&E staining. Slides were imaged using the Aperio Scanscope (Leica). Apoptosis was measured by TUNEL labeling as per manufacturer's protocol using the In-Situ Cell Death detection kit (Roche, 11684795910).

Western blot. Following an overnight recovery, islets were lysed in lysis buffer [150 mM NaCl, 50 mM Tris/Cl pH 7.4, 0.5% sodium deoxycholate, 0.1% SDS, 1% triton, 1x complete protease inhibitor (Roche), 1x PhosSTOP (Sigma-Aldrich)] followed by brief sonication. Protein concentrations were determined using Pierce BCA Protein Assay Kit (Thermo Fisher Scientific). Proteins were heat-denatured at 65°C for 10 min in NuPAGE LDS sample buffer (Thermo Fisher Scientific), resolved by SDS PAGE, and transferred to PVDF membranes (Bio-Rad). The membranes were incubated overnight at 4°C with antibodies prepared in 2% BSA (Sigma). The antibodies used are as follows: α-Tubulin (Santa Cruz sc-5286); SEL1L (Abcam ab78298 and homemade); HRD1 (Proteintech 13473-1-AP); LC3 (Cell signaling 2775); HSP90 (Santa Cruz sc-13119); IRE1α (CST 3294S); PERK (Cell Signaling 3192); Proinsulin (Novus NB100-73013); RTN3 (Bethyl A302-60A); FAM134B (Proteintech 21537-1-AP); RFP (Proteintech 6G6); Insulin (Bio-Rad, 5330-0104G); CLIMP63 (Proteintech 16686-1-AP) and RTN4 (Proteintech 10740-1-AP). Secondary antibodies were goat anti-rabbit IgG-HRP, anti-mouse IgG-HRP and anti guinea pig IgG-HRP (1:5,000; BioRad).

Pulse labeling. Islets were washed in prewarmed Met/Cys-deficient RPMI medium and then pulse-labeled with ^{35}S-amino acids (Tran^{35}S label) for 30 min at 37°C. Labeled islets were lysed in RIPA buffer (25 mM Tris, pH 7.5, 100 nM NaCl, 1% Triton X-100, 0.2% deoxycholic acid, 0.1% SDS, 10 mM EDTA) containing 2 mM NEM and a protease inhibitor cocktail and sonicated. Lysates were normalized to trichloroacetic acid-precipitable counts, and immunoprecipitated with guinea pig polyclonal anti-insulin and protein A-agarose overnight at 4°C. Immunoprecipitates were washed and analyzed by reducing 4-12% Bis-Tris NuPAGE gel, followed by phosphorimaging, and bands quantified with ImageJ software.

Immunofluorescent staining (Islet). Paraffin embedded pancreas sections were deparaffinized in xylene and rehydrated using graded ethanol series (100%, 90%, 70%) followed by rinse in distilled water. Antigen retrieval was performed by boiling the slides in sodium citrate or EDTA for 15 minutes. Sections were then incubated in a blocking solution (5% donkey serum, 0.03% TritonX-100 in PBS) for 1 hour at room temperature and with primary antibodies overnight at 4°C in a humidifying chamber. Following primary antibodies were used: Insulin (Bio-Rad, 5330-0104G; 1:500), Glucagon (Cell Signaling 2760s; 1:200), p62 (MBL PM045; 1:500), KDEL (Novus Biologicals NBP1-97469; 1:500), BiP (Abcam ab21685), GM130 (Abcam ab169276), Proinsulin (Novus Biologicals NB100-73013; 1:100), Aldh1a3 (Novus Biologicals, NBP2-15339; 1:100), MafA (Novus Biologicals, NBP1-00121; 1:100). Next day, following three washes with PBST (0.03% Triton X-100 in PBS), slides were incubated with respective Alexa Fluor conjugated to secondary antibodies (Jackson ImmunoResearch; dilution 1:500) for 1 h at room temperature followed by mounting with Vectashield mounting medium containing DAPI (Vector Laboratories, H-1500). The images of stained sections were captured using Nikon A1 Confocal Microscope at the University of Michigan Microscopy, Imaging and Cellular Physiology Core. Colocalization analysis was done using Image J software using Coloc2 algorithm.

Immunofluorescent staining (Cells). COS-7 cells were plated on coverslips and transfected with siRNA or plasmid as previously described (1). Twenty-four hours after DNA transfection, cells were fixed and stained with primary antibodies at 4 °C overnight. Following primary antibodies were used: anti-Myc (Santa Cruz Biotechnology, SC-40,1:100), anti-BiP (Abcam, ab21685, 1:200). Cells were then washed and incubated with fluorophore-conjugated secondary antibodies for 30 min in the dark at room temperature. Coverslips were mounted with ProLong Gold mounting medium with DAPI (Thermo Fisher, Cat# P36931) and imaged using the Zeiss LSM 780 confocal microscope.

Immunogold Staining. Islets were fixed in perfusion fixative (EMS 1224SK). For some experiments, islets were treated with 100 mM bafilomycin for 2 hours and transferred to fixation media. Samples were processed by the core and ultra-thin sections (70 nm) were placed in nickel grid. The grids were first quenched in 80 mM glycine and incubated in blocking solution (EMS 25599) for 30 min at room temperature and with primary antibodies overnight at 4°C in a humidifying chamber. Following primary antibodies were used: BiP (Abcam ab21685; 1:50) and

Proinsulin (Novus NB100-73013; 1:50). Next day, following washes with PBS, grids were incubated with colloidal gold antibodies (Jackson ImmunoResearch; dilution 1:20) for 1 hour at room temperature. Grids were then post fixed in 0.5% glutaraldehyde and contrasted using 0.5% uranyl acetate. Images were then taken using JEOL electron microscope at the University of Michigan Microscopy Core.

CRISPR/Cas9-based gene editing. Generation of SEL1L and HRD1-deficient HEK293T cells was previously described (2).

FIB SEM Imaging

Mice were sedated with isoflurane and perfused with 10 ml saline followed by 40 ml of fixative solution (4% PFA, 2.5% glutaraldehyde in 0.1 M cacodylate buffer). Pancreas was isolated and fixed overnight at 4°C. After fixation, samples were submitted to Michigan Microscopy Core for FIB SEM processing. Tissues were embedded in Durcupan ACM resin (Electron Microscopy Science). After polymerization, tissue blocks were mounted onto SEM pins with silver epoxy and then sputter coated with approximately 10 nm of carbon to find the region of interest. Samples were analyzed using a FEI Helios Nanolab 650 Dual Beam at the Michigan Center for Materials Characterization (University of Michigan). Cells of interest were located using back-scattered electron imaging at 10 kV accelerating voltage. Once a region of interest was identified, a platinum rectangle was deposited on a 5 × 5 µm region by SEM at 10 kV and 0.8 nA, followed by a protective deposition by FIB of carbon using 30 kV and 0.23 nA. Rough trenches around the region were cut by FIB using 30 kV and 9.3 nA beam current. The sample face was polished using 2.5 nA and 0.79 nA beam currents. The block was sequentially cut and imaged to obtain an image stack for tomographic reconstruction using the Auto Slice and View G3 V1.3 software. FIB cuts were made using 30 kV accelerating voltage and 80 pA beam current, while image of the block face was done using the SEM at 2 kV, set in backscatter mode. The field of view of the SEM and the slice thickness were set to produce 5 nm cubic voxels. The precise dimensions obtained are as follows: volume of 208 μm^3, comprising of 724 images for WT; 373 μm^3 with 1102 images for $Sel1L^{Ins1}$; 246 μm^3 with 1044 images for $Atg7^{Ins1}$ and 280 μm^3 with 1232 images for DKO β-cells. The resulting stack of images was aligned and reconstructed using Avizo v.9.3 (Thermo Fisher Scientific).

Single Cell Sequencing Data Analysis.

The published islet single-cell RNA seq data (GEO accession: GSE137785) were processed as previously described and cells were clustered using the Seurat package (version 4.1.0) (3) in the R environment. The "Autophagy Activity" calculated was using the normalized and scaled transcript counts for an autophagy induction gene list defined by Bordi et al. (4). For KEGG enrichment analysis, genes up-regulated in *Sel1L*^{Ins1} β-cells compared to wildtype were identified as having mean expression levels greater than 1.25-fold in *Sel1L*^{Ins1} with a false discovery rate (FDR) cutoff at 0.05. These genes were mapped to the KEGG pathway database and over-representation for each KEGG pathway was calculated using Fisher's Exact test. The FDR was calculated by comparing pseudo-bulked transcript counts for each gene between wildtype and *Sel1L*^{Ins1} β-cells and P values were calculated using the quasi-likelihood F-test implemented in the R package edgeR (5) (version 3.36.0) and were adjusted based on Storey's FDR approach (6).

References

1. Chen YJ, Knupp J, Arunagiri , Haataja L, Arvan P and Tsai B. PGRMC1 acts as a size selective cargo receptor to drive ER-phagic clearance of mutant prohormones. *Nat Comm*. 2021;12(1):5991
2. Shi G, Somlo D, Kim GH, Prescianotto-Baschong C, Sun S, Beuret N, et al. ER-associated degradation is required for vasopressin prohormone processing and systemic water homeostasis. J Clin Invest. 2017;127(10):3897-912.
3. Hao Y, Hao S, Andersen-Nissen E, Mauck WM, 3rd, Zheng S, Butler A, et al. Integrated analysis of multimodal single-cell data. *Cell.* 2021;184(13):3573-87 e29.
4. Bordi M, De Cegli R, Testa B, Nixon RA, Ballabio A, and Cecconi F. A gene toolbox for monitoring autophagy transcription. *Cell death & disease.* 2021;12(11):1044.
5. Robinson MD, McCarthy DJ, and Smyth GK. edgeR: a Bioconductor package for differential expression analysis of digital gene expression data. *Bioinformatics.* 2010;26(1):139-40.
6. Storey JD, and Tibshirani R. Statistical significance for genomewide studies. *Proc Natl Acad Sci U S A.* 2003;100(16):9440-5.

Supplemental figures and legends:

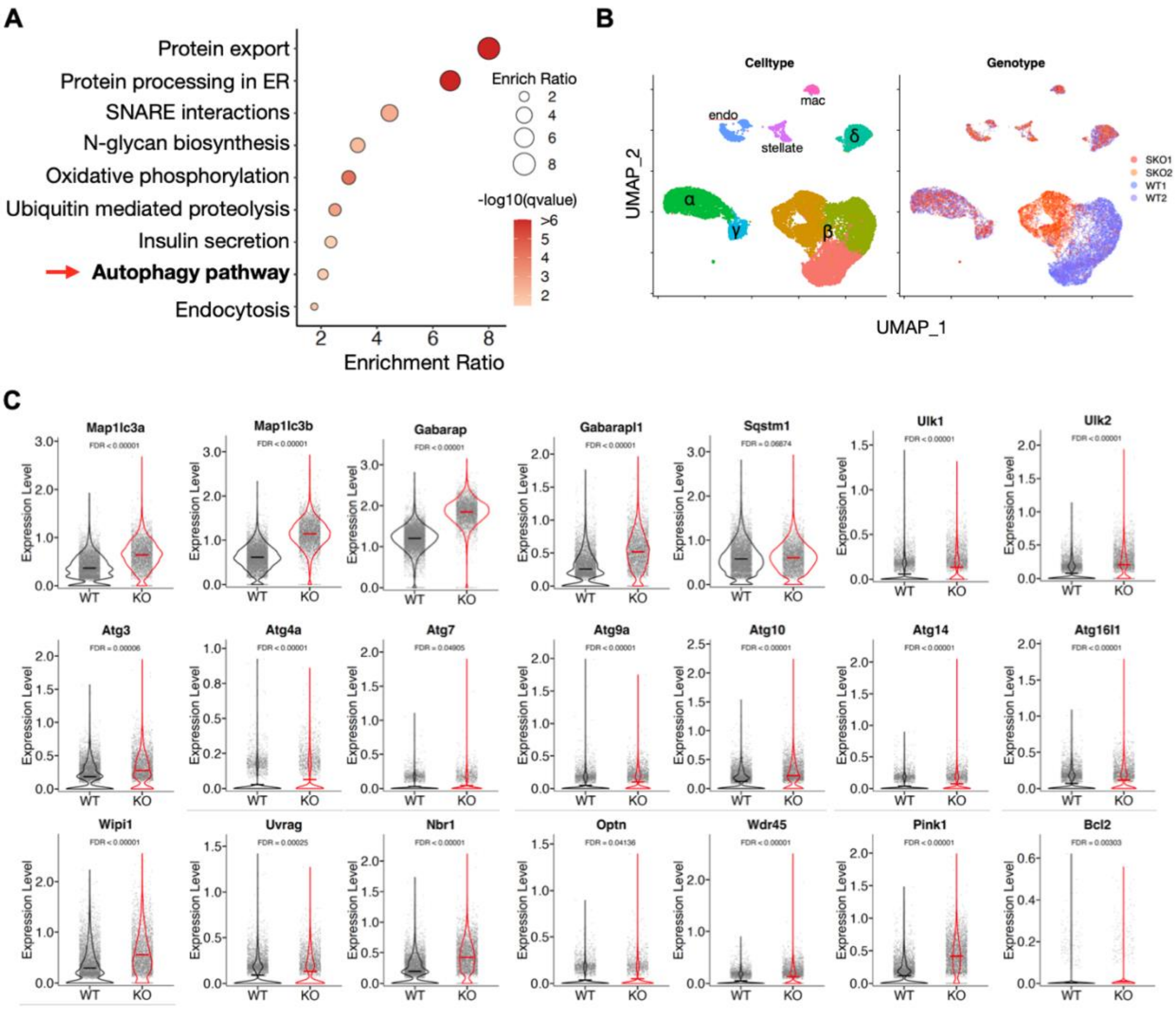

Figure S1. Basal autophagy is activated in ERAD-deficient β-cells. (A) Dot plot showing enriched gene ontology (GO) terms in *Sel1L^{Ins1}* beta cells. The 9 GO processes with the largest gene ratios are plotted in the order of gene ratio. The size of the dots represents the number of genes in the significantly enriched gene list associated with the GO term and the color of the dots represent the P-adjusted values. **(B)** UMAP plots of islets from WT and *Sel1L^{Ins1}* mice indicating cell type (left) and genotype (right), SKO indicates islets from *Sel1L^{Ins1}* mice. **(C)** Violin plots of reported autophagy-induced genes are shown, the bar represents the mean value. The false discovery rate (FDR) value is calculated using the quasi-likelihood F-test implemented in the R package edgeR for pseudo bulked transcript counts for each gene between the two genotypes and adjusted based on Storey's FDR approach.

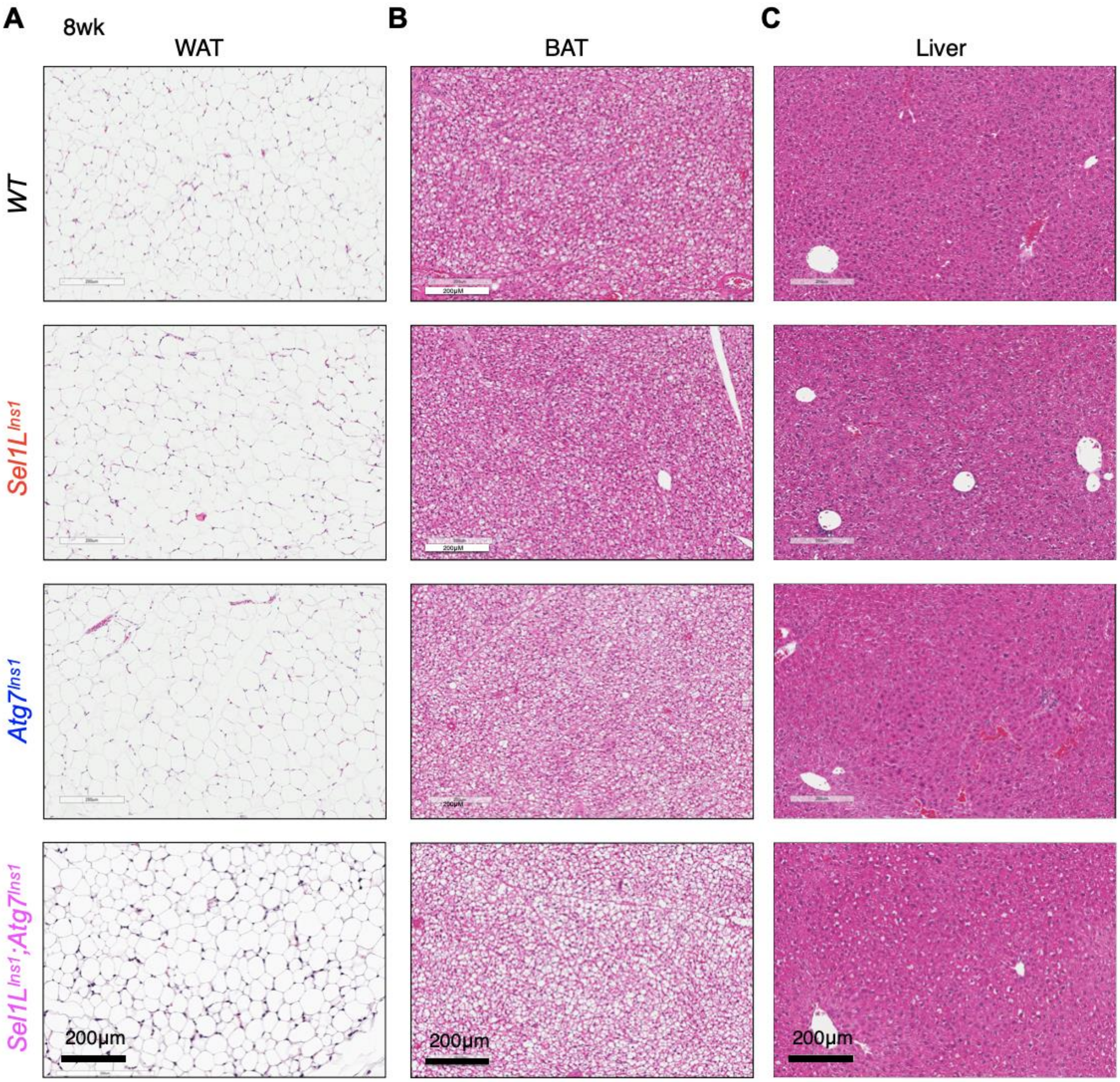

Figure S2. Comparable histological morphologies in peripheral tissues. (A-C) H&E staining of white adipose tissue (WAT), brown adipose tissue (BAT) and liver in the indicated genotypes at 8 wks of age.

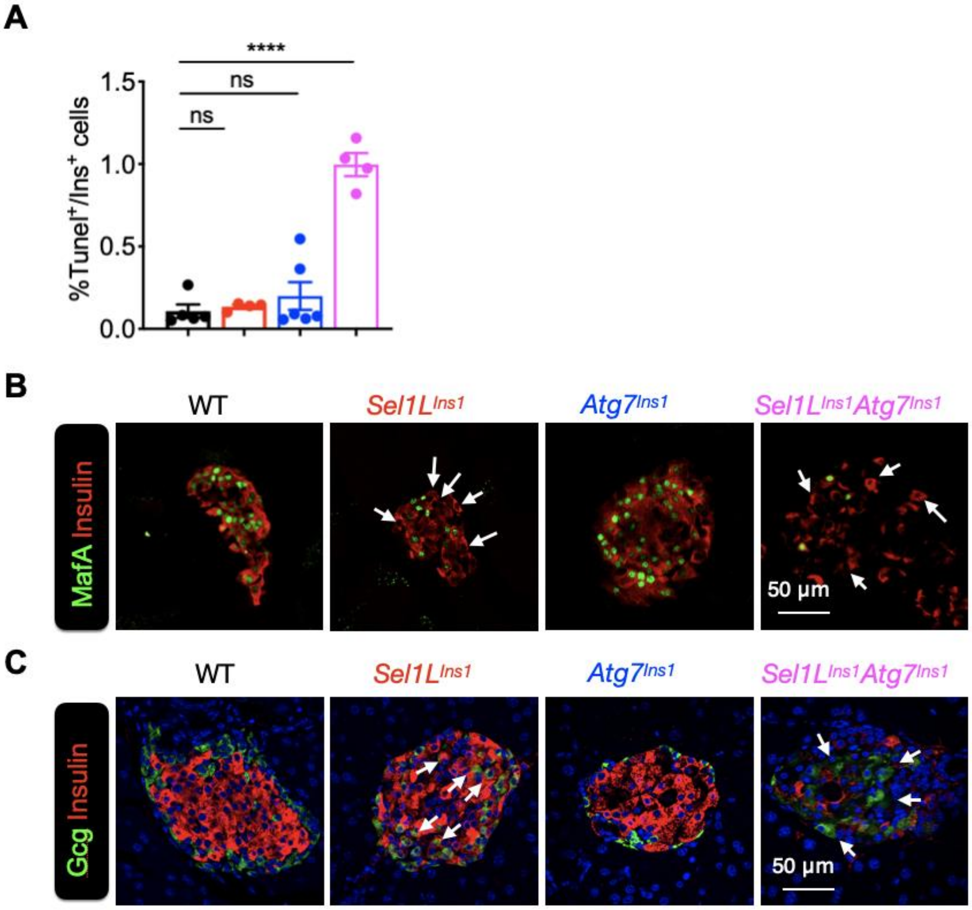

Figure S3. Synergistic effect of ERAD and autophagy on β-cell survival and identity. **(A)** Quantification of TUNEL positive β-cells (representative images shown in Fig. 2G, n = 4-6 per group, n.s., not significant, ****$P < 0.0001$, one-way ANOVA followed by Tukey's post hoc test, error bar indicates s.e.m.). **(B-C)** Representative confocal images of (B) MafA and insulin (arrow indicates MafA negative insulin positive β-cells) (C) insulin and glucagon (arrow indicates centrally located glucagon positive cells) in 8-week-old cohorts.

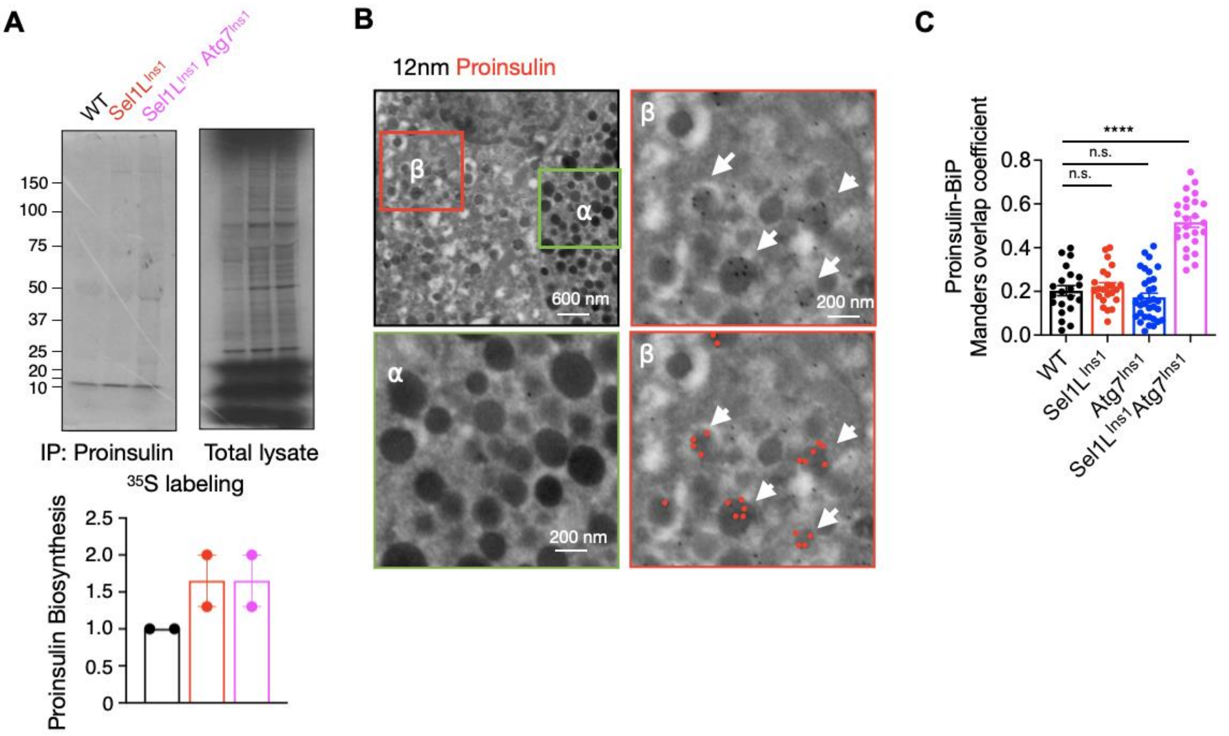

Figure S4. Normal rate of proinsulin biosynthesis in β cells lacking SEL1L (and ATG7) and immunogold specificity for proinsulin. (A) Pulse-chase and immunoprecipitation of proinsulin followed by SDS-PAGE in primary islets pulse-labeled with ^{35}S-methionine/cysteine for 30 min, with quantitation shown on the bottom (two repeats). **(B)** Specificity of proinsulin antibody is shown. α and β cells are indicated. Immunogold labeling of proinsulin was observed only in β-cells and not in α-cells. Arrow indicates proinsulin staining in the granules in beta cells. **(C)** Proinsulin-positive cells were examined for extent of proinsulin co-localization with the BiP (ER) using Manders' method using ImageJ software analyzed with 'Coloc2' algorithm. Each point indicates individual pancreatic ß-cells. *****P* < 0.0001, one-way ANOVA, error bar indicates s.e.m.

128

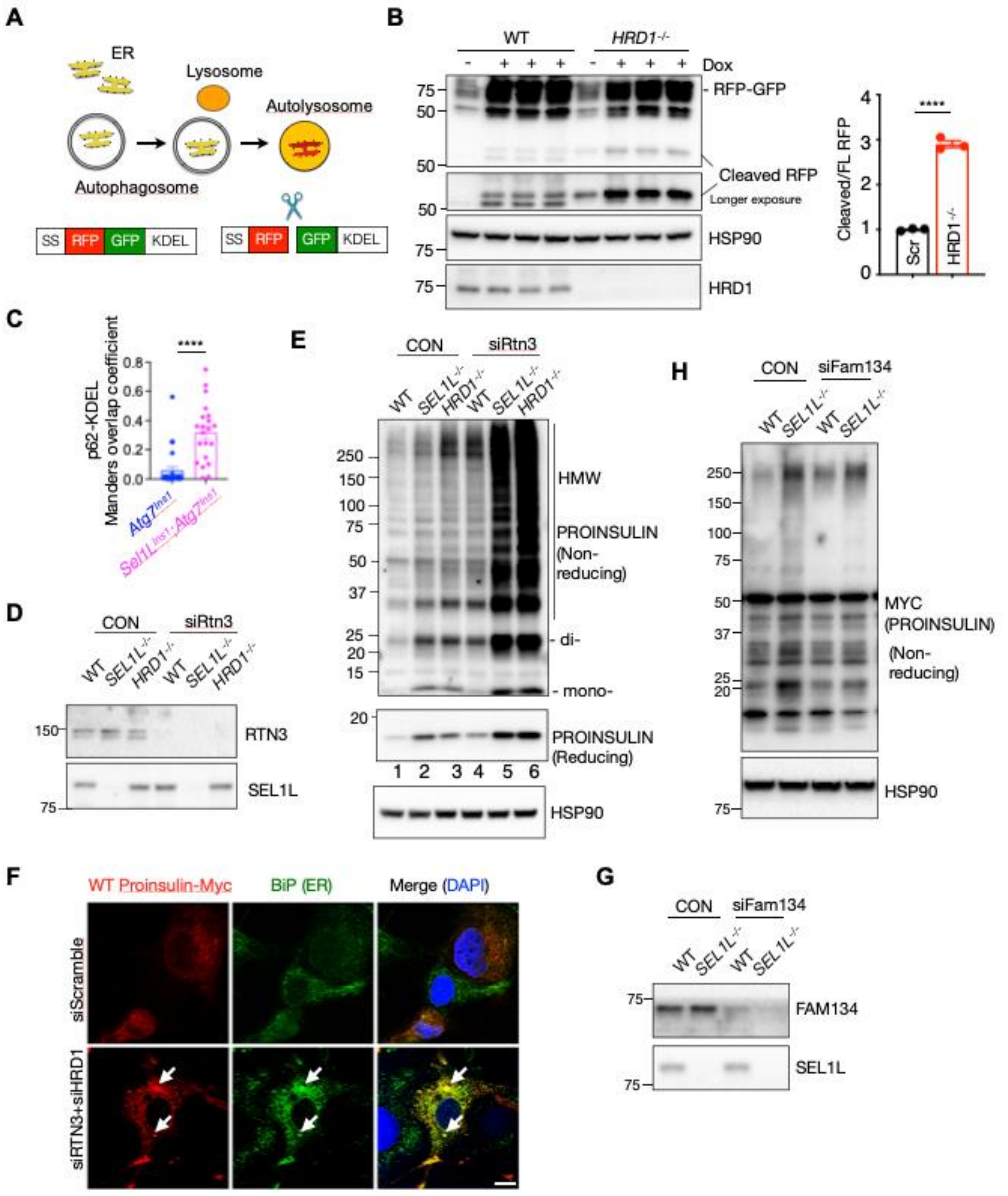

Figure S5. ERAD deficiency enhances ER-phagy in β-cells. (A) Schematic representation of the ER-phagy reporter ssRFP-GFP-KDEL. The reporter is cleaved in the lysosome to yield RFP. SS, signal sequence. **(B)** Western blot analysis of cleaved and full-length RFP in WT and HRD1$^{-/-}$ HEK cells transfected with ER-phagy reporter. **(C)** p62-positive cells were examined for extent of p62 co-localization with the KDEL (ER) using Manders' method using ImageJ software analyzed with 'Coloc2' algorithm. Each point indicates individual pancreatic β-cells. **(D-E)** Representative immunoblot analysis of WT Proinsulin under nonreducing (–β-ME) and reducing (+β-ME) SDS-PAGE in control, Sel1L and Hrd1 knockout HEK293T cells co-transfected with control or Rtn3 siRNA and with myc-tagged WT proinsulin. **(F)** Co-labeling of Myc (Proinsulin) and BiP in Cos7 cells treated with Scrambled (top) or Rtn3 and Hrd1 siRNA (bottom), over

expressing wild type proinsulin. Arrow indicates large punctate staining of proinsulin that colocalizes with BiP. Scale bar is 10µm. **(G-H)** Representative immunoblot analysis of WT Proinsulin under nonreducing (–β-ME) SDS-PAGE in control, Sel1L and Hrd1 knockout HEK293T cells co-transfected with control or Fam134 siRNA and with myc-tagged WT proinsulin. Two independent repeats. Values are shown as mean ± SEM. ***$P < 0.001$, ****$P < 0.0001$, Student’s t-test.

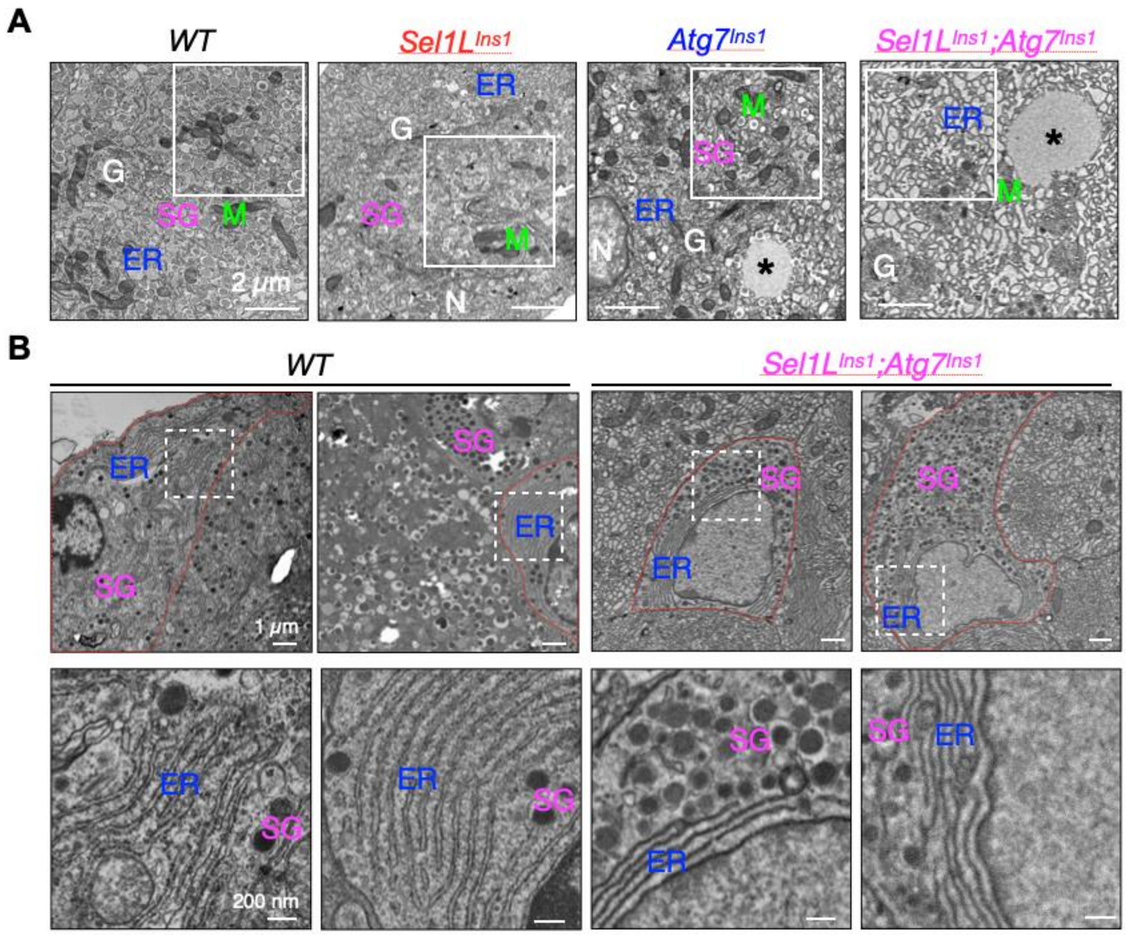

Figure S6. ERAD and ER-phagy synergistically re-shape organellar network in β cells. (A) Transmission electron microscopy (TEM) of pancreatic islets from WT, *Sel1L*^{Ins1}, *Atg7*^{Ins1} *and Sel1L*^{Ins1}*;Atg7*^{Ins1} mice. Scale bar, 1 µm. Asterisks indicate aggregate. Zoomed images are shown in Fig 5A. **(B)** TEM of pancreatic islets from *Sel1L*^{Ins1}*;Atg7*^{Ins1} mice with non-β-endocrine cells outlined in red. Boxed area shows the ER, zoomed in the lower panel. ER, Endoplasmic Reticulum; M, Mitochondria; G, Golgi apparatus; N, Nucleus; SG, Secretory Granules.

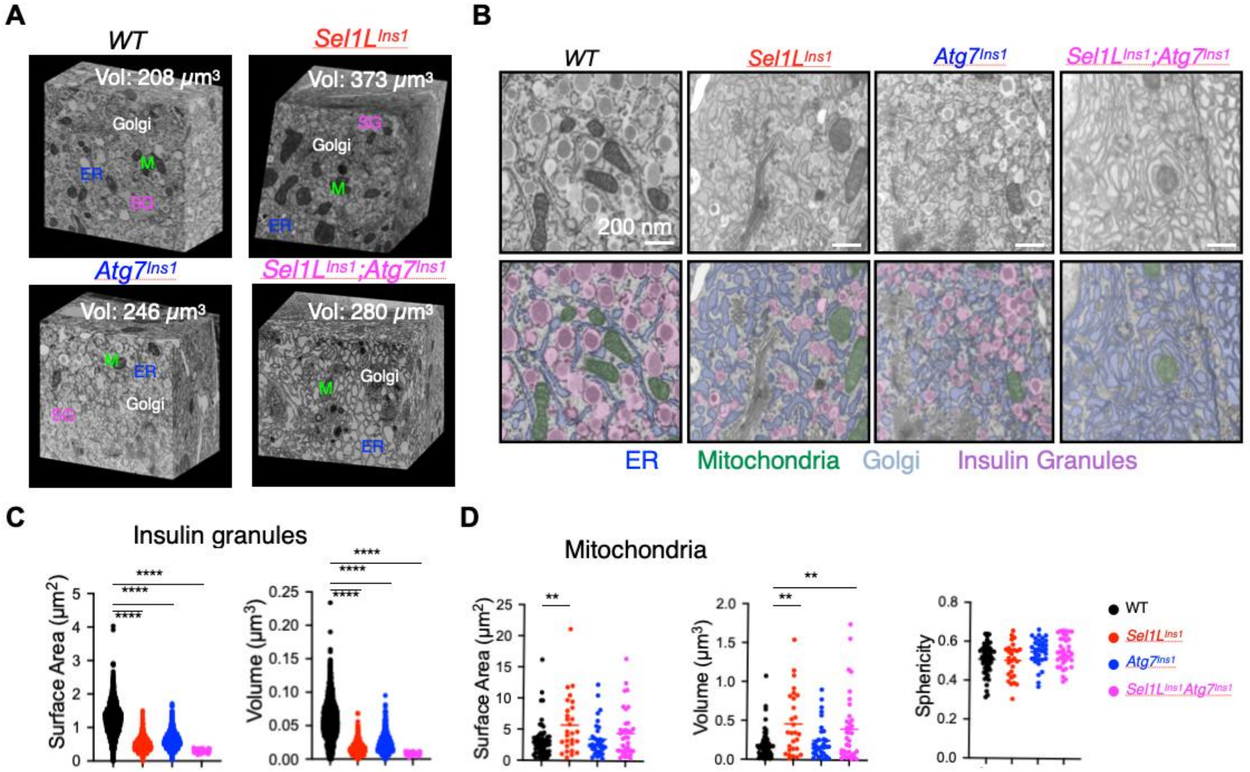

Figure S7. ERAD and ER-phagy synergistically re-shape organellar network in β cells. (A) Three-dimensional reconstruction of FIB-SEM images derived from β-cells from indicated genotypes. Volume is indicated. ER Endoplasmic Reticulum, M Mitochondria, SG Secretory Granules. **(B)** Machine learning based segmentation of ER, Golgi, mitochondria and insulin granules; scale bar, 200 nm. **(C-D),** Quantification of FIB-SEM data. Surface area and volume of insulin granules (C) and mitochondria (D) are shown for indicated genotypes. Each dot represents individual granules or mitochondria. **P < 0.01, ***P < 0.001, ****P < 0.001; one-way ANOVA.

131

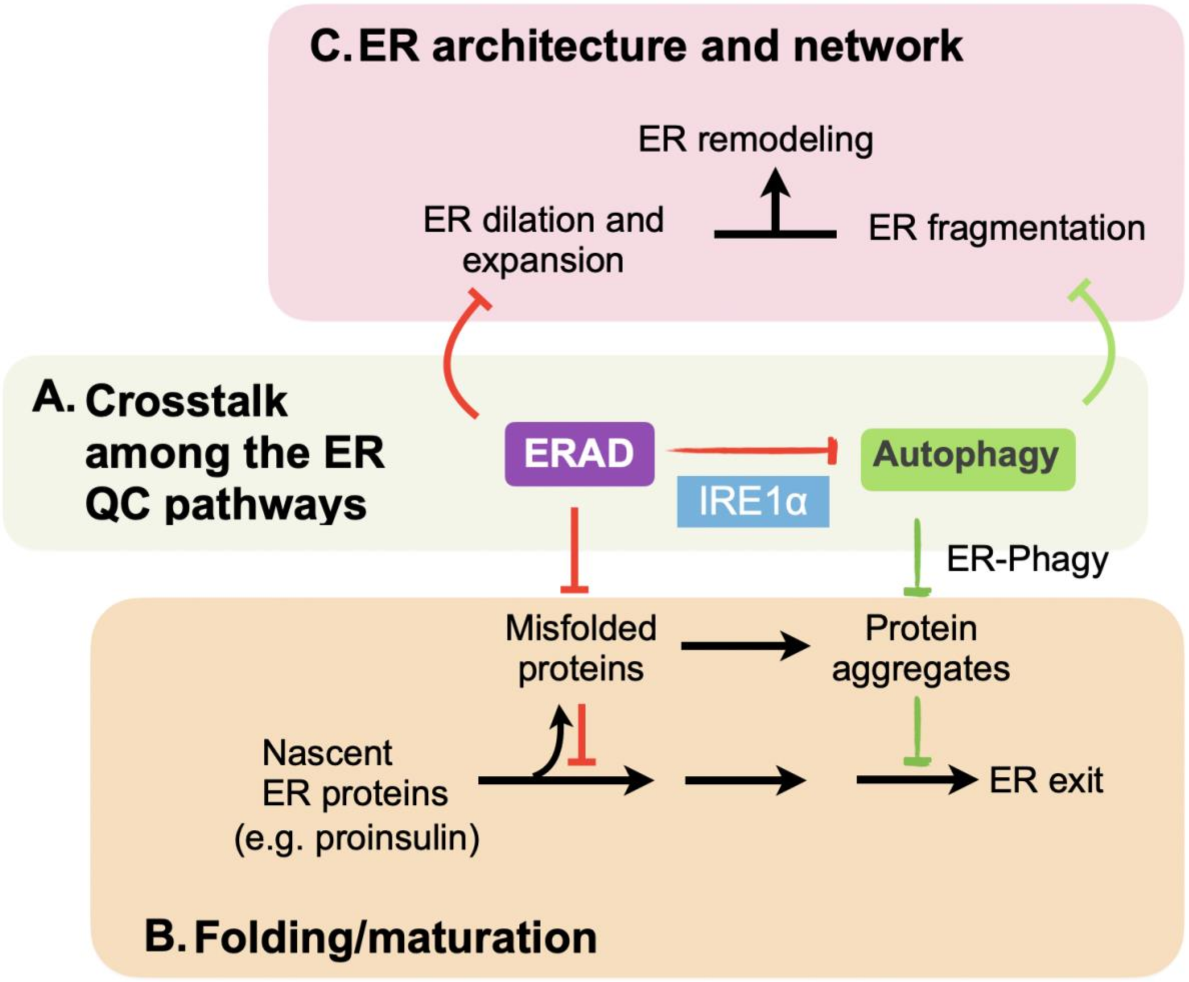

Figure S8. Proposed synergism between ERAD and autophagy/ER-phagy in β-cells. (A) Crosstalk among the ER QC pathways. **(B-C)** Synergism of ERAD and autophagy on **(B)** protein folding and maturation and **(C)** ER remodeling.

Supplemental video legends

Video 1. 3D FIB-SEM image series and animation showing reconstruction of endoplasmic reticulum, mitochondria, Golgi apparatus and insulin granules from *WT* β cell. Serial SEM images (724 sections, 5 nm/interval) of β cell from *WT* mice. Endoplasmic reticulum is shown in cyan, mitochondria in green, Golgi apparatus in grey and insulin granules in magenta.

Video 2. 3D FIB-SEM image series and animation showing reconstruction of endoplasmic reticulum, mitochondria, Golgi apparatus and insulin granules from $Sel1L^{Ins1}$ β cell. Serial SEM images (1102 sections, 5 nm/interval) of β cell from $Sel1L^{Ins1}$ mice. Endoplasmic reticulum is shown in cyan, mitochondria in green, Golgi apparatus in grey and insulin granules in magenta.

Video 3. 3D FIB-SEM image series and animation showing reconstruction of endoplasmic reticulum, mitochondria, Golgi apparatus and insulin granules from $Atg7^{Ins1}$ β cell. Serial SEM images (1044 sections, 5 nm/interval) of β cell from $Atg7^{Ins1}$ mice. Endoplasmic reticulum is shown in cyan, mitochondria in green, Golgi apparatus in grey and insulin granules in magenta.

Video 4. 3D FIB-SEM image series and animation showing reconstruction of endoplasmic reticulum, mitochondria, Golgi apparatus and insulin granules from *DKO* β cell. Serial SEM images (1232 sections, 5 nm/interval) of β cell from *DKO* mice. Endoplasmic reticulum is shown in cyan, mitochondria in green, Golgi apparatus in grey and insulin granules in magenta.
